# Development of a dietary index based on the Brazilian Cardioprotective Nutritional Program (BALANCE)

**DOI:** 10.1186/s12937-018-0359-5

**Published:** 2018-05-04

**Authors:** Jacqueline Tereza da Silva, Ângela Cristine Bersch-Ferreira, Camila Ragne Torreglosa, Bernardete Weber, Renata Bertazzi Levy

**Affiliations:** 1 Department of Nutrition, School of Public Health, University of São Paulo, Avenida Doutor Arnaldo, 715, São Paulo, SP 01246904 Brazil; 2 Research Institute, Hospital do Coração (HCor), Rua Abílio Sorares, 250, 12° andar, São Paulo, SP 04005-000 Brazil; 3 Department of Preventive Medicine, School of Medicine, University of São Paulo, Avenida Doutor Arnaldo, 455, São Paulo, SP 01246903 Brazil

**Keywords:** Dietary assessment, Nutritional index, dietary patterns, Reliability and validity, Cardiovascular disease

## Abstract

**Background:**

The diet of the Brazilian Cardioprotective Nutritional Program (BALANCE) classifies food into four groups and sets the daily amount to be consumed. The dietary approach of BALANCE is different from other dietary recommendations; therefore, it is not possible to use existing dietary indexes (DI) to assess patient’s adequacy to BALANCE diet. For this reason, it is important to develop a specific dietary index based on BALANCE diet.

This study aims to describe the development of the BALANCE DI, evaluate its internal consistency, construct and content validity and population characteristics associated with the index.

**Methods:**

We analyzed baseline data from the BALANCE randomized clinical trial (https://www.clinicaltrials.gov/; NCT01620398). The four food groups of the diet were adopted as index components. Points ranging from 0 to 10 were given to each index component. Internal consistency was evaluated by correlation coefficients between total score and component scores, as well as Cronbach’s Alpha. Content and construct validity were assessed by checking how nutrients are associated with the index and if the index could distinguish between groups with known differences in diet, respectively. Crude and adjusted linear regression analyses were performed to evaluate population characteristics associated with the index.

**Results:**

The analysis included 2044 subjects (58.6% men). The average of the total index was higher among women (*p* < 0,05). The components of the index showed low correlations with each other. The correlations between each individual component with the total index were > 0.40. Cronbach’s alpha coefficient was 0.66. High scores in the index were inversely associated (p < 0,05) with energy, total fat, monounsaturated fat (MUFA) and cholesterol; they were positively associated (p < 0,05) with carbohydrates and fiber. Hypertensive men and diabetic women had higher scores, while male smokers had lower scores.

**Conclusions:**

The BALANCE DI showed reliability and construct validity similar to other DI. It also detected characteristics of individuals that are associated with higher or lower index scores.

## Background

Dietary habits are important modifiable risk factors for cardiovascular diseases (CVD), the first cause of death and burden of disease worldwide and in Brazil [1, 2]. The World Health Organization (WHO) emphasizes that a healthy diet, able to prevent CVD, is influenced by many complex interactions (income, food availability and affordability, individual preferences and beliefs, cultural traditions, geographical, environmental, social and economic factors) [3]. To take these complex interactions into account, the choice of foods that compose a healthy and cardioprotective diet should be regionalized.

Brazil has a variety of food rich in cardioprotective components such as fiber, vitamins and bioactive compounds that could be used to promote cardiovascular protection and minimize the burden of CVD. The Brazilian Cardioprotective Nutritional Program (BALANCE) is an educational intervention aimed at improving the consumption of foods available in the country with a potential cardioprotective role. The Brazilian Ministry of Health has been assessing the effectiveness of the BALANCE program in a multicentric and randomized clinical trial since 2013 [4].

The dietary component of the BALANCE program ranked foods in groups represented by the colours of the Brazilian flag. As green is the most abundant colour in the flag, the items included in the green group should be eaten more often. Yellow is the second most abundant flag colour, thus the advice is to eat foods in the yellow group in moderation. Blue represents foods that should be consumed in smaller quantities. Ultra-processed foods are not recommended in the BALANCE diet and are represented by red, which is not in the Brazilian flag. Details about the food classification criteria have been published elsewhere [4].

Several dietary indexes (DI) have been developed to study associations between dietary patterns and CVD in different populations [5–10]. A DI combines the many aspects of a nutritional recommendation or guideline and uses them as the items of the index. These aspects are generally the amount of nutrients, foods or food groups to be eaten in each period of time (day/week/month). Since the dietary aspects of the BALANCE program are different from other cardioprotective dietary patterns, it is not possible to use existing DI to assess the role of the BALANCE diet on cardiovascular protection. We therefore aim to describe the development of the BALANCE DI, to assess its internal consistency, construct and content validity, and to evaluate the population characteristics associated with the index.

## Methods

### Study population

To develop the DI we used data from the BALANCE study (https://www.clinicaltrials.gov/; NCT01620398), a multicentric randomized clinical trial. The trial aims to assess the effects of the Brazilian Cardioprotective Nutritional Program in the secondary prevention of cardiovascular diseases. Between March 2013 and January 2015, 2535 adults over 44-years-old and with a personal history of cardiovascular disease were enrolled in the trial and followed-up until December 2017 in one of the 35 sites in Brazil. Details of the trial procedures are given elsewhere [4]. The 2044 individuals who had full dietary data at baseline were included in the present analysis, which was approved by the School of Public Health/University of São Paulo Research Ethics Committee.

### Data collection and description of variables

Data were collected in the 35 sites by trained researchers following standardized procedures. Details of the data collection are given in the BALANCE research protocol, available on request from the authors [4].

Dietary intake was assessed from a single 24-h recall collected by trained interviewers who asked for a detailed description of all foods and drinks consumed the day before the interview, following an adaptation of the Multiple-Pass Method steps [11]. A photographic album of food portion sizes was available to help with the estimation of quantities of food consumed [12]. The food intake data was recorded in Nutriquanti Software using household measures and then converted to grams. The software calculated the energy and nutrient amount using information from different food composition tables, including the Brazilian Food Composition Table and the USDA Food Composition Table. All the 24-h recalls that ranged over less than 1000 kcal or more than 3000 kcal were double-checked. Discrepant home measures, such as “zero tablespoon” and “150 teacups” were also double-checked. Records with less than the 1st or greater than the 99th percentile of energy were excluded [13]. The relative participation of macronutrients in the diet was expressed as the percentage of total energy intake. Cholesterol, sodium and fiber were presented as milligrams or grams per each 1000 kcal ingested.

Height was measured twice with wall-mounted stadiometers (0.5 cm precision) with the subject barefoot in a standing position. Weight was collected twice with calibrated scales (100 g precision) with the subject barefoot and wearing light clothes. Waist circumference (WC) was measured twice with an anthropometric tape in the midpoint between the lowest rib and the iliac crest. The average of the measurements was used. If the measurements differed more than 0.5 kg for weight, 0.5 cm for height and 1 cm for WC, the entire procedure was repeated until the difference between the two new measures was lower than the established cut-off. Body mass index (BMI) was calculated as weight divided by the square of height (kg/m^2^) and classified according to age. If age ≤ 59 years-old, underweight = BMI < 18.5 kg/m^2^, normal weight = BMI ≥ 18.5 kg/m^2^ and < 25 kg/m^2^, overweight = BMI ≥ 25 kg/m^2^ [14]. If age ≥ 60 years-old, underweight = BMI < 23 kg/m^2^, normal weight = BMI ≥ 23 kg/m^2^ and < 28 kg/m^2^, overweight = BMI ≥ 28 kg/m^2^ [14, 15].

A questionnaire was applied to investigate the personal history of CVD and smoking status. Diagnoses of diabetes, hypertension and dyslipidemia were self-reported.

### The dietary component of the BALANCE program

The dietary component of the BALANCE program [4] is designed to meet the nutritional recommendations proposed by the Brazilian Cardiovascular Guidelines [16–20]. To implement these recommendations on nutritional advice, we compiled a list of cardioprotective foods, based on a set of qualitative criteria defined by nutrition experts considering the most relevant aspects of Brazilian nutritional guidelines: a) no added sugar; b) low-calorie content; c) lack of nutrients that increase cardiovascular risk (cholesterol, saturated fat, and sodium); and d) presence of cardioprotective nutrients (antioxidants and dietary fiber). The result of the compilation was a list consisting of low-fat yoghurt and milk, fruits and vegetables, and beans cooked with garlic, onion, soybean oil (up to 1%) and refined salt (up to 0.5%).

Energy, saturated fat, cholesterol and sodium densities [21] of foods in the list were evaluated to create cutoff values for food classification into the green, yellow and blue groups. The maximum density values of the list were 1.11 kcal per gram, 0.01 g of saturated fatty acid per gram, 0.04 mg of cholesterol per gram, and 2.01 mg of sodium per gram. These values were applied in the food categorization as follows: foods with all 4 density values equal to or less than the cutoffs were assigned to the green group (vegetables, fruits, beans, low-fat milk and yoghurt); foods with one or two densities above the cutoffs were assigned to the yellow group (rice, bread, pasta, oats, couscous, nuts, vegetable oil); foods with three or four nutrient densities above the cutoffs were categorized into the blue group (meat, fish, cheese, eggs).

Finally, the BALANCE diet contains a red group. The cutoffs were not adopted as criteria for assigning foods in this group. Instead, foods lacking beneficial components such as vitamins, antioxidants and dietary fiber and known as sources of trans fats, refined sugar, artificial sweeteners, and preservatives – mainly ultra-processed foods - compose the red group.

### The BALANCE dietary index

We created the BALANCE DI considering the aspects of the existing DI that are used to analyze associations between dietary patterns and cardiovascular disease [7, 22–25]. The BALANCE Program recommends a number of servings in each food group depending on the energy level, as shown in Table 1. Energy need was calculated multiplying current body weight by 20 kcal in overweight or 25 kcal in normal weight or 32 kcal in underweight participants [17, 26]. Individuals with calculated need below 1400 kcal were set to 1400 kcal, those above 2400 kcal were set to 2400 kcal. Intermediate values were assessed accordingly to mathematical approximation rules. For example, if the calculated need is 1758 kcal, the nutritionist must assign into the 1800 kcal, the closest value.

**Table 1:** BALANCE recommendation of servings per day in each food group according to the energy-level^a^

	Number of servings per day					
BALANCE food groups	1400	1600	1800	2000	2200	2400
	Kcal	Kcal	Kcal	Kcal	Kcal	Kcal
Green (vegetables, fruits, beans and legumes, low-fat milk)	9	11	11	12	14	16
Yellow (rice, bread, pasta, oats, couscous, nuts, vegetable oil)	6	7	9	10	11	13
Blue (meat, fish, cheese, eggs)	2	2	3	3	4	4
Red (ultra-processed foods)	0	0	0	0	0	0

BALANCE = Brazilian Cardioprotective Nutritional Program

^a^This content was published in the BALANCE research protocol, is reproduced with permission and available on request from the authors [14]

The index has four components corresponding to the BALANCE food groups. The scoring criteria are summarized in Table 2 and were made a priori, based on the BALANCE principles and recommendations and the population distribution of consumed servings. Each component was given a score ranging from 0 (worst) to 10 (best), therefore the total score ranges from 0 to 40.

**Table 2:** Scoring criteria for the BALANCE dietary index, mean score (95% confidence interval) among men and women with a history of cardiovascular disease

Index component (BALANCE food group)	Criteria for 0 point^*^	Criteria for 10 point*	Score for men	Score for women
Green (vegetables, fruits, beans and legumes, low-fat milk)	0	≥ the recommendation	4.49 (4.34; 4.65)	4.55 (4.36; 4.73)
Yellow (rice, bread, pasta, oats, couscous, nuts, vegetable oil)	50% > or < the recommendation	= the recommendation	3.50 (3.30; 3.69)	3.52 (3.29; 3.74)
Blue (meat, fish, cheese, eggs)**	≥ 2 beyond the recommended	≤ the recommendation	5.20 (4.95; 5.46)	6.09 (5.80;6.38)
Red (ultra-processed foods)**	≥ 4	≤ 0	5.37 (5.14; 5.61)	5.90 (5.64; 6.16)
Total score (range)**	0	40	18.56 (18.11; 19.02)	20.05 (19.54; 20.56)

BALANCE = Brazilian Cardioprotective Nutritional Program

*Servings/d between the minimum and maximum criteria were given the scores proportionally

**Statistical differences between means *p* < 0.05 (Mann-Whitney test)

A score of zero is given to the green group when there is no consumption and a score of 10 when the consumption is equal or higher than the recommendation. A score of 10 is assigned to the yellow group when the number of servings is exactly that recommended. The minimum score is assigned to the yellow group when the number of servings is 50% over or below the recommendation. The blue and red groups received a reverse score (higher intakes are given lower scores). In the blue group, the maximum score is assigned to the number of servings equal to or lower than the recommendation and the minimum when there are at least two servings above the recommendation. Finally, in the red group, a score of 10 is assigned when there is no consumption, and a score of 0 is given to 4 or more reported servings. Those participants consuming intermediate amounts were scored proportionally.

To calculate the score, we classified all food items reported in the 24-h recalls into the BALANCE food groups and defined the size of a serving. Then, we calculated the number of servings consumed and the corresponding score for each component. Finally, we summarized the component values to obtain the total score.

Because the BALANCE DI was developed to assess the consistency with the BALANCE Program dietary component, we decided to follow the rationale of the Program and use the BALANCE food groups as the items of the score. The foods in the green group have low energy, saturated fat, cholesterol and sodium density and higher levels of cardioprotective compounds [4]. Furthermore, the consumption of these foods is low in the Brazilian population [27] and we aim to improve the intake of such foods. For this reason, we did not assign a penalty for overconsumption and used a monotonic function to score this component (higher levels of intake received higher scores).

As the yellow group is the major provider of energy intake, we believe the consumption should be neither higher nor lower than the recommendation, to maintain the energy balance. Hence, the scoring system has two directions with intakes under or above the recommendation being penalized.

The blue group is mainly responsible for the saturated fat, cholesterol and sodium intake and received a reverse score (higher intakes get lower scores). The red group is not recommended in the BALANCE diet and therefore also gets a reverse score. For these items in the index there is no clear scientific evidence determining what amount of intake should receive the minimum score of zero. To improve discriminatory power and the ability to detect differences in the population, we determined, as the cutoff, an approximate value of the 75th percentile of the population intake distribution. A similar strategy has been adopted in previous studies [28].

### Statistical analysis

The scores of the overall index and its components were described in mean and 95% confidence interval (95% CI) for males and females and differences were assessed by Mann-Whitney tests. Subsequent analyses were performed separately by sex because the scores differed among men and women.

To evaluate the internal consistency of the index we analyzed the inter-item correlation matrix, and calculated the Spearman rank correlation coefficients between the components and total index. We also estimated the Cronbach’s coefficient alpha. Values higher than 0.70 indicated reliability (internal consistency).

To assess construct validity, we checked if the index could distinguish between groups with known differences in diet (men and women). To assess the content validity, we verified if the index was associated with key nutrients in an expected direction, dividing it into quartiles and calculating the mean and 95% CI of energy and selected nutrient intakes in each quartile. The fourth quartile indicated the highest adequacy with the BALANCE, whereas the first quartile indicated the lowest adequacy with the diet. Tests for linear trends were performed to compare the population intakes across the quartiles.

The population characteristics associated with the index were assessed by linear regression, crude and adjusted for age, BMI, waist circumference, hypertension, dyslipidemia, diabetes and smoking status. All the analyses were conducted in STATA software, version 12, and statistical significance was defined as *p* < 0.05.

## Results

The analysis included 2044 subjects with a mean (standard deviation) age of 63 [9] years. Among males (58.6% of the population), 88.3% were hypertensive, 78.5% dyslipidemic, 41.9% diabetic, 63.3% former smoker and 7.4% current smoker and the mean (SD) of BMI and WC was, respectively, 28.5 (4.4) Kg/m^2^ and 101.1 (11.3) cm. Among females, 93.2% were hypertensive, 79.0% dyslipidemic, 45.7% diabetic, 41.6% former smoker and 8.0% current smoker and the mean (SD) of BMI and WC was, respectively, 29.8 (5.5) Kg/m^2^ and 97.9 (12.9) cm.

Table 2 describes the criteria for minimum and maximum score in each component of the BALANCE DI and shows the mean (95% CI) for the components and total score among men and women. The results differed between sexes, with females having higher scores in the total index, and in the blue and red components (Table 2). The findings for the green and yellow groups were similar in both sexes.

The correlations among BALANCE DI components and total index are shown in Table 3. The components of the index had low correlations among themselves (*r* < 0.10). The blue and red groups showed similar correlations with the total index (*r* = 0.61 and *r* = 0.62, respectively) and were more correlated with the total index than the other components, indicating that their variance contributes more to the total score. Nevertheless, the green and yellow groups do add important information to the total score as they show correlations higher than 0.40. The Cronbach’s coefficient alpha of the BALANCE dietary index was 0.66.

**Table 3:** Correlation between BALANCE diet components and total index

	Green group	Yellow group	Blue group	Red group	Total index
Green group	1				
Yellow group	0.0113	1			
Blue group	0.0096	0.0053	1		
Red group	0.0883	0.0430	0.0892	1	
Total index	0.4033	0.4594	0.6131	0.6157	1

BALANCE = Brazilian Cardioprotective Nutritional Program. Green group = vegetables, fruits, beans and legumes, fat free milk and yogurt. Yellow = rice, bread, pasta, oats, couscous, nuts, vegetable oil. Blue = meat, fish, cheese, eggs. Red = ultra-processed foods

Table 4 presents the mean (95% CI) of energy and selected nutrient intakes across quartiles of the BALANCE index among men and women. Higher scores were inversely associated with intakes of energy, total fat, MUFA and cholesterol and positively associated with intakes of carbohydrate and fiber. Among males, there was no difference in the consumption of proteins across the quartiles of the score and among females, the intake of protein was higher in the 4th quartile. No difference was observed in the intake of polyunsaturated fat and sodium.

**Table 4:** Energy and selected nutrient intake (mean and 95% confidence interval) across quartiles of the BALANCE dietary index among men and women with a history of cardiovascular disease

Dietary component	MEN				WOMEN			
	1	2	3	4	1	2	3	4
n	300	299	299	299	211	212	212	212
BALANCE index range	0; 13.31	13.32; 18.33	18.34; 24.79	24.80; 38.62	0; 14.51	14.52; 20.54	20.55; 25.59	25.61; 39.59
Energy, Kcal	1851 (1777; 1926)	1629 (1560; 1697)	1453 (1396; 1509)	1345 (1293; 1398)	1498 (1416; 1579)	1279 (1206; 1351)	1213 (1140; 1287)	1169 (1116; 1221)
Protein, % Kcal	19.2 (18.4; 20.0)	19.2 (18.3; 19.8)	19.2 (18.5; 19.9)	18.3 (17.7; 18.9)^1^	17.5 (16.6; 18.5)	18.7 (17.9; 19.7)	17.8 (17.1; 18.6)	18.8 (18.1; 19.6)
Carbohydrate, % Kcal	48.0 (46.6; 49.3)	50.8 (49.6; 52.0)	54.6 (53.4; 55.8)	58.7 (57.5; 59.9)	51.3 (49.8; 52.7)	54.1 (52.7; 55.6)	57.2 (55.6; 58.8)	59.3 (57.8; 60.7)
Total fat, % Kcal	32.9 (31.9; 33.8)	30.8 (29.9; 31.8)	27.2 (26.3; 28.1)	24.9 (24.0; 25.8)	31.7 (30.5; 32.9)	28.2 (27.0; 29.4)	26.9 (25.7; 28.2)	24.1 (23.0; 25.2)
Saturated, % Kcal	10.9 (10.5; 11.3)	8.4 (9.4; 10.1)	8.4 (8.0; 8.8)	7.1 (6.8; 7.5)	11.0 (10.5; 11.5)	9.2 (8.7; 9.7)	8.4 (7.9; 8.7)	7.2 (6.8; 7.7)
Monounsaturated, % Kcal	9.1 (8.7; 9.5)	9.8 (8.2;9.0)	7.7 (7.3; 8.1)	6.9 (6.6; 7.2)	8.8 (8.3; 9.2)	7.5 (7.0; 7.9)	7.3 (6.8; 7.8)	6.9 (6.3; 7.4)
Polyunsaturated, % Kcal	7.5 (7.1; 7.9)	7.9 (7.5; 8.4)	7.4 (7.1; 7.8)	7.3 (7.0; 7.7)^1^	6.9 (6.4;7.3)	7.3 (6.8; 7.8)	7.1 (6.6;7.6)	6.7 (6.3; 7.1)^1^
Cholesterol, mg/1000 Kcal	162 (149; 174)	141 (130; 152)	133 (121; 145)	111 (102; 120)	147 (134; 160)	130 (119; 141)	113 (101; 125)	103 (94; 112)
Sodium, mg/1000 Kcal	1922 (1845; 2000)	1818 (1752; 1885)	1860 (1800; 1920)	2090 (1762; 2417)^1^	1760 (1678; 1852)	1873 (1748; 1999)	1798 (1711; 1884)	1777 (1690; 1863)^1^
Fiber, g/1000 Kcal	10.1 (9.6; 10.6)	12.3 (11.8; 12.9)	14.5 (13.8; 15.1)	17.8 (17.0; 18.6)	10.2 (9.5; 10.9)	13.2 (12.4; 13.9)	15.0 (14.0; 16.0)	17.2 (16.2; 18.2)

BALANCE = Brazilian Cardioprotective Nutritional Program. ^1^The *P*-value for linear trend was not significant (≥ 0,05). All others were significant, *P* < 0,05

Table 5 presents population characteristics associated with the index. In general, males with hypertension and females with diabetes had higher scores in the index, while male smokers had lower scores. On average, the adjusted analyses showed hypertensive men having 1.77 higher scores than non-hypertensives, male current smokers had 2.13 lower scores compared with males who never smoked and diabetic women had 1.56 higher scores than non-diabetics.

**Table 5:** Association between the BALANCE dietary index and characteristics of men and women with history of cardiovascular diseases

	MEN				WOMEN			
Population characteristics	Crude		Adjusted^a^		Crude		Adjusted^a^	
	Coefficient	95% CI	Coefficient	95% CI	Coefficient	95% CI	Coefficient	95% CI
Age, years (continuous)	0.06	0.01; 0.11	0.04	−0.01;0.10	0.05	−0.01; 0.11	0.04	−0.022; 0.10
BMI, kg/m2 (continuous)	− 0.09	− 0.20; 0.01	− 0.02	− 0.24; 0.20	− 0.08	− 0.17; 0.01	0.02	− 0.16; 0.20
Waist circumference, cm (continuous)	− 0.04	− 0.08; 0.00	− 0.04	− 0.12; 0.05	− 0.04	− 0.08; 0.00	− 0.06	− 0.13; 0.02
Hypertension (no = 0, yes = 1)	1.77	0.35; 3.18	1.86	0.41; 3.32	−2.38;	−2.26; 1.78	− 0.37	−2.46; 1.73
Dyslipidemia (no = 0, yes = 1)	− 0.20	−1.30; 0.91	− 0.53	−1.67; 0.61	− 0.18	− 1.43; 1.07	− 0.39	− 1.68; 0.90
Diabetes (no = 0, yes = 1)	0.37	−0.55; 1.29	0.40	− 0.54; 1.34	1.18	0.16; 2.20	1.56	0.48; 2.63
Smoking status (never smoked = 0)								
Former smoker	0.01	− 1.00; 1.03	0.01	− 1.01; 1.03	0.30	− 1.67; 2.27	−0.12	− 1.21; 0.96
Current smoker	−2.53	−4.41; − 0.66	−2.13	−4.03; − 0.22	0.64	− 1.30; 2.58	−0.15	− 2.14; 1.84

BALANCE = Brazilian Cardioprotective Nutritional Program

*BMI* Body mass index

^a^Adjusted for age, BMI, waist circumference, hypertension, dyslipidemia, diabetes and smoking status

## Discussion

BALANCE DI was developed to reflect the recommendations of the dietary component of the BALANCE Program. The index showed reasonable internal consistency, construct and content validity and detected population characteristics associated with higher or lower scores.

The ability to detect known differences in the population corroborates the construct validity of a DI [25]. It is well known that women in general eat more healthily than men and the BALANCE DI found variations across sex, with women having higher scores in the total index. This finding is consistent with previous studies assessing different diet indexes, which also observed women having a more favorable diet [25, 28–30]. The higher consumption of fruits and vegetables by women is usually the reason they obtain better scores in DI [28]. However, our study showed no differences in the consumption of fruits and vegetables (green food group) across sex, but found lower consumption of meat (blue food group) and ultra-processed foods (red food group) among women. The BALANCE DI also detected differences in the diet between smoker and non-smoker males. On average, smokers had lower scores than non-smokers.

The Cronbach’s coefficient alpha measures the internal consistency and values higher than 0.70 indicate accepted reliability. Similar to other studies which found alphas between 0.6 and 0.7 [25, 31], the alpha coefficient for the BALANCE DI was 0.66. According to Guenther and colleagues [25], given the complex and multidimensional construct of DI and because individuals do not fully meet and do not fully fail to meet all the dietary standards used in the indexes, an alpha coefficient slightly lower than 0.70 is expected. The low inter-item correlation (*r* < 0.10) and correlations ≥0.40 between each item and total index strengthen the reliability of the index.

The BALANCE DI showed association with energy and key nutrients in an expected direction, because it was inversely associated with intakes of energy, total fat, MUFA and cholesterol and it was positively associated with intakes of fiber and carbohydrate. These findings are consistent with previous diet quality indexes for Swedish, Australian and American population without cardiovascular disease [9, 29, 32], except the 14-point Mediterranean diet adherence screener which was inversely associated with carbohydrate and positively associated with MUFA [33]. In concordance with our findings, Mendes and colleagues found that higher intakes of total fat were significantly associated with lower index scores in the Healthy Eating Index for the Brazilian population [30].

The lower levels of MUFA in the highest quartile of the index were expected because the sources of MUFA in western diets are usually the same as the sources of saturated fat [34] and they should be consumed in small quantities in the BALANCE diet. Furthermore, the BALANCE diet encourages the intake of foods available and accessible in Brazil, which are not beneficial sources of MUFA, contrary to the reality of the Mediterranean region [4, 35].

Nevertheless, the relative participation of carbohydrate increases as the score in the index increases. This could be explained by the lower relative participation of total fat in the higher levels of the score. Because the amount of fiber was also positively associated with the index, another explanation for the higher quantities of carbohydrate in the top levels of the index is the high intake of fruits in the BALANCE diet (data not shown), which has, as a benefit, elevated amounts of vitamins and bioactive compounds with a cardioprotective role [36].

The higher percentage of protein among women in the 4th quartile of the index is an unexpected result. We have two possible explanations for that. First, the macronutrients are presented as a percentage of energy, which means that if the percentage of one macronutrient changes the others will consequently change. Thus, the higher amount of protein could be explained by the lower relative participation of total fat in the highest quartile of the index. Second, an increase in protein intake from green group foods (beans and legumes, fat-free milk and yoghurt) may also have led to this result.

We were not able to detect associations between the index and the intake of sodium. A possible explanation is the fact that the sodium intake was estimated from 24-h recalls, which is not a robust instrument for estimating sodium intake. The gold standard method to estimate sodium intake is the 24-h urinary excretion, but these data were not available. A previous study estimated sodium intake from semi-quantitative food frequency questionnaire and was also unable to detect an association between the DI and the intake of sodium [27].

In our study, we observed lower index scores among males smoking and higher scores among males with hypertension and females with diabetes. Previous studies did not find associations between hypertension and diabetes diagnosis and conformance with DI [32, 37], but found associations between DI and BMI, WC and smoking status [29, 32, 33]. It is important to highlight that these studies included patients without previous CVD, while BALANCE included patients in the secondary prevention. Thus, subjects from BALANCE trial may have already received nutritional advice from dieticians, doctors, and nurses before being enrolled in the trial, making them aware of the eating choices they should make. This is a possible explanation for having hypertensive and diabetics subjects with better index scores.

The present study has limitations. The diagnoses of diabetes, hypertension and dyslipidemia were self-reported. However, this is unlikely to have added bias to the study because participants are in the secondary prevention of cardiovascular disease, which requires them to take some medications daily. By the type of taken medication (antihypertensive, for example) patients and interviewers know their health condition.

Although the dietary data were collected by trained interviewers following standardized procedures and were submitted to quality control, the usual intake could not be estimated because we had only one 24-h recall. Usually intake is estimated with at least two 24-h recalls to soften the effects of within-person variance in the assessment of the diet-disease relationship. The present study described the development of a dietary index based on the BALANCE diet and did not assess diet-disease relationships. Furthermore, a single 24-h recall can characterize the average consumption of a group and can be used when the sample size is large (higher than 1000) [38].

The number of servings in the blue group is an upper limit, i.e. if the recommendation is two servings then two servings or less should be eaten, while foods in the red group are not supposed to be part of the diet. Blue and red groups are given a reverse score and there is no clear scientific evidence or logical value clarifying how high an intake should be to deserve the minimum score of zero. Thus, we decided to use data-driven cut-offs to avoid giving the maximum or minimum score to a large proportion of the population. The decision to use data-driven cut-offs could compromise the external validity of the index. It is also important to note that our findings are derived from a Brazilian population aged 45 years or older with a personal history of major cardiovascular events (stroke, coronary heart disease, peripheral vascular disease) and the BALANCE dietary index can characterise the diet of similar populations.

## Conclusion

In this manuscript, we described the development of BALANCE DI and assessed some aspects of its validity, as well as population characteristics associated with higher or lower scores.

Regarding the internal consistency, BALANCE DI showed performance similar with other DI. The construct validity was corroborated by the ability to discriminate groups with known differences in the diet (males/females). The positive association with the consumption of carbohydrate and fiber, probably reflecting the intake of fruits, and the inverse association with intakes of energy, total fat, MUFA and cholesterol reinforces the content validity. Concerning population characteristics, we observed that diabetic women and hypertensive men were more likely to have higher index scores while smoking males were more likely to have lower scores.

The BALANCE DI has several potential uses: evaluation of the BALANCE Program dietary intervention; monitoring trial participants’ compliance with the diet during the follow-up period; assessment of the association between the BALANCE diet and major CVD events; prediction of CVD risk (future). Before applying the index for all these purposes, we suggest studies with longitudinal designs to assess the validity of the index against health outcomes and its performance in detecting the relationship between overall diet and health.

## Abbreviations

BALANCE: Brazilian Cardioprotective Nutritional Program
BMI: Body mass index
CI: Confidence interval
CVD: Cardiovascular disease
DI: Dietary index
MUFA: Monounsaturated fatty acids
SD: Standard deviation
USDA: United States Department of Agriculture
WC: Waist circumference
WHO: World Health Organization
